# Appetite‐regulating hormones and anthropometric indicators of infants according to the type of feeding

**DOI:** 10.1002/fsn3.1381

**Published:** 2020-01-09

**Authors:** Edgar Vásquez‐Garibay, Alfredo Larrosa‐Haro, Elizabeth Guzmán‐Mercado, Nelly Muñoz‐Esparza, Samuel García‐Arellano, Francisco Muñoz‐Valle, Enrique Romero‐ Velarde

**Affiliations:** ^1^ Instituto de Nutrición Humana Universidad de Guadalajara Guadalajara Mexico; ^2^ Instituto de Investigación en Ciencias Biomédicas Universidad de Guadalajara Guadalajara Mexico; ^3^ Nuevo Hospital Civil de Guadalajara Dr. Juan I. Menchaca Guadalajara Mexico

**Keywords:** anthropometric indicators, appetite‐regulating hormones, infants, type of feeding

## Abstract

It has been accepted that satiety‐ and appetite‐stimulating hormones play a role in the regulation of food intake and body composition during and after the lactation stage. Therefore, the purpose was to demonstrate that serum appetite‐regulating hormones in infants differ according to anthropometric indicators and type of feeding. In a nonrandom cohort study, 169 mother–newborn dyads whose pregnancy and birth were attended at the Hospital Civil de Guadalajara were enrolled. According to the type of feeding, infants were classified as full breastfeeding (FBF), partial breastfeeding (PBF), and infants receiving human milk substitutes (HMS). Serum concentrations of ghrelin (pg/ml), leptin (ng/ml), peptide YY (pg/ml), and glucagon‐like peptide‐1 (GLP‐1) (pM) were measured. Anthropometric measurements including weight, length, cephalic, arm circumference, tricipital, and subscapular skinfolds were obtained. Weight/age, weight/height, height/age, and BMI *Z*‐score indexes were estimated. We performed one‐way ANOVA, unpaired Student's *t* test, post hoc Tukey test, and Pearson correlation tests. The ANOVA comparison of the three feeding types showed significant differences in most anthropometric indicators (*z*‐scores), especially between infants receiving FBF versus HMS and particularly on indicators of adiposity; no differences were observed in length and cephalic circumference *z*‐scores at 8th and 16th weeks. Further, significant correlations were found between most of the adiposity indicators with ghrelin, leptin, and GLP‐1, especially in infants who received FBF. There were differences in anthropometric and body composition parameters among infants receiving FBF, PBF, and HMS. There were significant correlations between body composition indicators with ghrelin, leptin, and GLP‐1 mainly in infants receiving FBF.

## Graphical abstract

This was a comparative and correlational analysis of a nonrandom cohort study of 169 infant/mother dyads. We measured appetite‐regulating hormones and performed anthropometry related to growth and body composition; the analysis was carried out according to the type of feeding. We found differences in body composition according to the type of feeding and correlations of anthropometric indicators with ghrelin, leptin, and GLP‐1, especially in infants who received full breastfeeding.

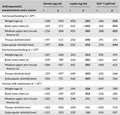

**Key messages**

- There are differences in anthropometric and body composition indicators among infants receiving full breastfeeding, partial breastfeeding, and human milk substitutes at 8 and 16 weeks of postnatal age.
- There are marked differences in serum concentration of appetite‐regulating hormones in infants, especially in the highest concentrations of hormones related to increased food intake and growth.
- There is a direct and often significant trend of linear correlations of most indicators of fat reserve with ghrelin, leptin, and GLP‐1, especially in infants who received full breastfeeding

## 1 INTRODUCTION

Being overweight or obese are the most frequent nutritional problems in schoolchildren, adolescents, and adults in Mexico (Hernández‐Ávila et al., 2016). This condition has disturbed most sectors of society because of its adverse effects on health and its magnitude, which demands that immediate action be taken to halt its progress (US Preventive Services Task Force et al., 2017). We all come into the world with our own genetic profile; however, there is a critical period, the first one thousand days of life, in which neonates, infants, and toddlers are particularly sensitive to interaction with the environment (Blumfield, 2015; Langley‐Evans, 2015). In this context, breastfeeding and then perceptual nutrition after the sixth month play a fundamental role (Mochizuki, Hariya, Honma, & Goda, 2017).

It has also been noted that the protective role of breastfeeding against the development of obesity could be partially explained by the presence of appetite‐regulating hormones (ARHs) in breastfed infants (Li, Magadia, Fein, & Grummer‐Strawn, 2012; Petridou et al.., 2005; Savino et al., 2008). However, the role of the system in regulating appetite in states of hunger/starvation and in the pathogenesis of overeating/obesity remains to be fully elucidated in humans (Farr, Li, & Mantzoros, 2016).

It is accepted that satiety‐ and appetite‐stimulating hormones play a role in the regulation of body composition by signaling satiety and energy reserves through hypothalamic receptors, during and after the lactation stage (Marić et al., 2014; Münzberg & Morrison, 2015). Satiety‐regulating hormones such as leptin, glucagon‐like peptide (GLP‐1), and peptide YY decrease food intake, promote satiety, decrease the desire to eat, and increase the metabolic rate (Breij, Mulder, van Vark‐van der Zee, & Hokken‐Koelega, 2017; Schueler, Alexander, Hart, Austin, & Larson‐Meyer, 2013). Therefore, the purpose of the study was to demonstrate that the serum concentration of leptin, ghrelin, glucagon‐like peptide‐1 (GLP‐1), and peptide YY in infants differs according to the type of feeding and their body composition.

## 2 METHODS

### 2.1 Design

This was a comparative and correlational analysis of a nonrandom cohort study. We identified mother–newborn dyads who were admitted to the Physiological Puerperium Ward in a shared room at the Nuevo Hospital Civil de Guadalajara. The criteria of inclusion, exclusion, and the estimation of the sample size are described elsewhere (Vasquez‐Garibay et al., 2019).

### 2.2 Dependent variables

Serum appetite‐regulating hormones: total ghrelin (pg/ml); leptin (ng/ml); peptide YY (pg/ml); and glucagon‐like peptide‐1 (GLP‐1) (pM). Anthropometric indicators: weight (g), length (cm), cephalic circumference (cm), arm circumference (cm), tricipital and subscapular skinfolds (mm), and *z*‐scores of weight/age, weight/height, length/age, cephalic circumference/age, and BMI.

### 2.3 Independent variables

Full breastfeeding (FBF); partial breastfeeding (PBF); and human milk substitutes (HMS) based on cow's milk.

### 2.4 Measuring instruments and techniques

After standardization of two observers (EGM, NME), the following anthropometric measurements were made (Frisancho, 1990):

#### 2.4.1 Weight

Infants were placed naked and without a diaper on the scale, taking care that the whole body remained inside the tray and was distributed evenly over the center of the tray. A weighing scale model 314 (SECA) was used to measure weight; the precision was 10 g.

#### 2.4.2 Length

The measurement was obtained by two observers with an infant bed model 416 (Seca). The infant was placed in the supine position, with the body aligned straight on the longitudinal axis of the infantometer so that the shoulders and hip remained in contact with the horizontal plane and the arms were on the sides of the trunk. The crown of the head touched the fixed base of the infantometer and was placed on the plane of Frankfort, that is, aligned perpendicular to the horizontal plane. One of the observers held both the head and the base of the infantometer. The other observer, with one hand, stretched the infant's legs, watching for the knees to be deflected, and with the other hand moved the movable base of the infantometer, so that a slight pressure (only slightly compressing the skin) was exerted on the heel of the infant free from any object. The foot remained at an angle of 90°. The measurement had an accuracy of 0.1 cm.

#### 2.4.3 Cephalic circumference

This measurement was made with a 6‐mm‐wide metal tape (Rosscraft®, USA). The tape was applied firmly around the head in the supraciliary region, so that the tape ran along the most prominent part of the frontal area and occipital protuberance.

#### 2.4.4 Mid‐upper arm circumference (MUAC)

The measurement was performed on the left arm. An observer bent and held the arm at a 90° angle to the forearm and marked half the distance from the acromion to the olecranon as a midpoint. Subsequently, the measurement was performed with the arm extended in the middle of the arm previously marked, using a metal tape model 201 of 6 mm wide. A tricipital skinfold (TSF) measurement was taken on the midpoint of the inner, rear surface of the previously marked arm; a subscapular skinfold (SSF) measurement was taken at the lower edge of the scapula. Both measurements were performed on the left side using a Lange skinfold caliper. The weight/age, height/age, weight/height, and BMI indices and cephalic circumference were estimated in the *Z*‐score using the WHO growth standard (WHO 2006).

The collection of blood samples and the assays for the determination of hormonal biomarkers are described elsewhere (Vasquez‐Garibay et al., 2019).

### 2.5 Fieldwork criteria and strategies

Once the mothers had signed the informed consent form, demographic, socioeconomic, and educational dietary variables were collected; the mothers were then contacted after a period of one month to ask them about the type of feeding they had chosen for their infants. Anthropometric measurements were performed during each of the 8 and 16 weeks appointments; at 16 weeks, a blood sample was taken from the infants. There were no significant differences in the general characteristics of the mother/infant dyads between study participants and mothers, who were not located by telephone at 4 weeks postpartum or those who declined to participate in the study.

### 2.6 Statistical analysis

The comparison of variances of the anthropometric data between the groups was performed with ANOVA for one factor and Tukey *post hoc* tests. The linear relationship between measurements and anthropometric indexes and the ARHs was performed with Pearson correlations. The level of significance considered for all statistical tests was <0.05. Calculations of the anthropometric indices were estimated by the WHO Anthro version 3.2.2. The statistical analysis was carried out with the SPSS 21 software.

## 3 RESULTS

The total sample at 8 and 16 weeks postpartum was 169 dyads: FBF (74), PBF (57), and HMS (38). The age of the infants in the three types of feeding was 8 ± 1 weeks in the first visit and 16 ± 1 weeks in the second visit. Tables 1, 2, 3 show the crude data and *z*‐scores of the anthropometric indicators of infants at 8 and 16 weeks of postnatal age. The ANOVA tests for the comparison of the three feeding types showed significant differences between them in most anthropometric indicators and their *z*‐scores except for *z*‐scores of length and cephalic circumference at 8 weeks of postnatal age and length and cephalic circumference (both in cm and *z*‐score) at 16 weeks of postnatal age. Crude anthropometric values and *z*‐scores of the indices showed significant differences between 8 and 16 weeks mainly between the FBF versus HMS and FBF versus PBF. In most measurements and indices, infants with FBF had z‐score values greater than those of the other two groups.

**Table 1:** Length and cephalic circumference and their *z*‐scores of 169 infants at 8 and 16 weeks of postnatal age according to the type of feeding: full breastfeeding (FBF), partial breastfeeding (PBF), and human milk substitutes (HMS). Comparison with one‐way ANOVA; Tukey *post hoc* test (significant values with *post hoc* tests are presented as footnotes)

Postnatal age	Anthropometric measurements and indices	FBF (*n* = 74)		PBF (*n* = 57)		HMS (*n* = 38)		*p*
		*x*	*SD*	*x*	*SD*	*x*	*SD*	
8 weeks	Length (cm)	57.0	1.6	57.1	1.6	56.1	1.9	**.012**
	Length/age (z)	−0.62	0.8	−0.61	0.8	−0.77	0.9	.577
	Cephalic circumference (cm)	38.7	1.1	38.5	0.9	38.2	1.1	**.021**
	Cephalic circumference (z)	−0.24	0.8	−0.35	0.7	−0.38	0.7	.581
16 weeks	Length (cm)	62.2	2.0	62.1	1.6	61.7	2.2	.445
	Length/age (z)	−0.62	0.9	−0.62	0.7	−0.74	0.9	.727
	Cephalic circumference (cm)	40.9	1.2	40.8	0.9	40.5	1.3	.214
	Cephalic circumference (z)	−0.38	0.9	−0.39	0.7	−0.55	0.8	.526

Note

Significant post hoc tests at 8 weeks. Length: FBF versus HMS, ***p* = .024**. PBF versus HMS, ***p* = .017**. Cephalic circumference: C: FBF versus PBF, ***p* = .015**.

**Table 2:** Anthropometric measurements and indices of 169 infants at 8 and 16 weeks of postnatal age according to the type of feeding: full breastfeeding (FBF), partial breastfeeding (PBF), and human milk substitutes (HMS). The comparison among groups was made with one‐way ANOVA; significant p values with Tukey *post hoc* tests are presented as footnotes

Postnatal age	Anthropometric measurements and indices	FBF (*n* = 74)		PBF (*n* = 57)		HMS (*n* = 38)		*p*
		*x*	*SD*	*x*	*SD*	*x*	*SD*	
8 weeks	Weight (g)	5,316	576	5,019	494	4,770	583	**<.001**
	Weight/age (z)	−0.33	0.8	−0.7	0.6	−0.93	0.8	**.001**
	Weight/length (z)	0.47	0.9	−0.24	0.9	−0.19	1.0	**<.001**
	Body mass index	16.3	1.3	15.4	1.2	15.1	1.2	**<.001**
	Body mass index (z)	0.01	0.9	−0.61	0.8	−0.67	0.9	**<.001**
16 weeks	Weight (g)	6,642	722	6,333	589	6,179	855	**<.001**
	Weight/age (z)	−0.31	0.9	−0.64	0.7	−0.82	0.9	**.008**
	Weight/length (z)	0.19	1.0	−0.32	0.8	−0.37	1.0	**.003**
	Body mass index	17.1	1.5	16.4	1.1	16.2	1.6	**<.001**
	Body mass index (z)	0.05	1.0	−0.44	0.7	−0.54	1.1	**.002**

Note

Significant post hoc test at 8 weeks. Weight: FBF versus PBF, ***p* = .008;** FBF versus HMS, ***p* < .001**. Weight/age: FBF versus PBF, ***p* = .041**, FBF versus HMS, ***p* = .001**. Weight/length: FBF versus PBF, ***p* < .001**. FBF versus HMS, ***p* = .004**. Body mass index: FBF versus PBF, ***p* < .001;** FBF versus HMS, ***p* < .001**. Z‐BMI: FBF versus PBF, ***p* = .001**. FBF versus HMS, ***p* = .001**. Significant post hoc tests at 16 weeks. Weight: FBF versus HMS, ***p* < .001**. Weigh/age: FBF versus HMS, ***p* < .001.** FBF versus PBF, *p* = .031. Body mass index: FBF versus HMS, ***p* < .001**. FBF versus PBF, ***p* = .007**. Z**‐**BMI: FBF versus HMS, ***p* = .006**. FBF versus PBF, ***p* = .012**.

**Table 3:** Arm anthropometric measurements and *z*‐scores and subscapular skinfold of 169 infants at 8 and 16 weeks of postnatal age according to the type of feeding: full breastfeeding (FBF), partial breastfeeding (PBF), and human milk substitutes (HMS). Comparison among groups with one‐way ANOVA; significant values with Tukey *post hoc* tests are presented as footnotes

Postnatal age	Anthropometric measurements and indices	FBF (*n* = 74)		PBF (*n* = 57)		HMS (*n* = 38)		*p*
		*x*	*SD*	*x*	*SD*	*x*	*SD*	
8 weeks	Medium upper arm circumference (cm)	12.0	0.88	11.7	0.50	11.4	0.80	**<.001**
	Medium upper arm circumference (z)	−0.63	1.11	−1.02	0.62	−1.40	1.00	**<.001**
	Triceps skinfold (mm)	8.8	1.67	7.8	1.40	8.1	1.56	**.001**
	Triceps skinfold (z)	1.80	1.46	0.95	1.27	1.14	1.42	**.002**
	Subscapular skinfold (mm)	8.4	1.59	7.5	1.59	7.8	1.52	**.002**
16 weeks	Medium upper arm circumference (cm)	13.0	0.96	12.6	0.6	12.4	1.1	**<.001**
	Medium upper arm circumference (z)	−0.62	0.92	−1.02	0.55	−1.09	1.00	**.005**
	Triceps skinfold (mm)	9.5	1.8	8.5	1.3	8.9	1.7	**.002**
	Triceps skinfold (z)	−0.13	1.15	−0.70	0.89	−0.34	0.99	**.008**
	Subscapular skinfold (mm)	8.6	1.9	7.8	1.5	8.0	1.9	**.05**
	Subscapular skinfold (z)	0.57	1.26	0.12	1.07	0.27	1.19	**.09**

Note

Significant post hoc test at 8 weeks. Medium upper arm circumference: FBF versus PBF, ***p* = .034**. FBF versus HMS, ***p* = .001**. Medium upper arm circumference (z): FBF versus PBF, ***p* = .034**. FBF versus HMS, ***p* = .001**. Triceps skinfold: FBF versus PBF, ***p* = .001**. FBF versus HMS, ***p* = .033**. Triceps skinfold (z): FBF versus PBF, ***p* = .002**, FBF versus HMS, ***p* = .048**. Subscapular skinfold: FBF versus PBF, ***p* = .002**. Significant post hoc tests at sixteen weeks. Medium upper arm circumference: FBF versus HMS, ***p* = .001.** FBF versus PBF, ***p* = .016**. Medium upper arm circumference (z): FBF versus HMS, ***p* = .025**. FBF versus PBF, ***p* = .015**. Triceps skinfold: FBF versus PBF, ***p* = .036**. FBF versus MHS, ***p* = .002**. Triceps skinfold (z): FBF versus PBF, ***p* = .006**. Subscapular skinfold: FBF versus PBF, ***p* = .048**.

Table 4 shows that ghrelin correlated inversely and significantly with SSF measurement in infants who received FBF, while leptin correlated directly and significantly with the five anthropometric indicators, especially weight/age, BMI, and MUAC. In addition, with FBF there was also a direct and significant correlation of GLP‐1 with four of the five anthropometric indicators, especially with MUAC. In those who received PBF, the leptin correlations with the anthropometric indicators were equally significant; there was no correlation of anthropometric indicators with ghrelin and with GLP‐1. In infants receiving HMS, the correlation profile of anthropometric indicators with leptin was similar although lesser significant than with FBF and PBF; however, with this type of feeding, there was no correlation with ghrelin or with GLP‐1. The peptide YY did not correlate with any anthropometric indicator in the three types of feeding.

**Table 4:** Correlation coefficients among the serum concentration of ghrelin, leptin, and glucagon‐like peptide (GLP‐1) with anthropometric measurements and *z*‐score indices in 157 4‐month‐old infants classified by the type of feeding: full breastfeeding, partial breastfeeding, and feeding with human milk substitutes

Anthropometric measurements and *z*‐scores	Ghrelin (pg/ml)		Leptin (ng/ml)		GLP−1 (pM/ml)	
	*r*	*p*	*r*	*p*	*r*	*p*
Full breastfeeding (*n* = 69^a^)						
Weight/age (z)	−.208	.093	.400	**.001**	.266	**.033**
Body mass index (z)	.165	.173	.461	**<.001**	.242	.054
Medium upper arm circumference (z)	−.216	.086	.455	**.001**	.288	**.022**
Triceps skinfold (mm)	−.197	.113	.256	**.045**	.191	.131
Subscapular skinfold (mm)	−.297	**.016**	.312	**.014**	.253	**.044**
Partial breastfeeding (*n* = 53^b^)						
Weight/age (z)	−.107	.460	.343	**.013**	.076	.591
Body mass index (z)	.039	.789	.466	**.001**	−.061	.667
Medium upper arm circumference (z)	−.006	.967	.461	**.001**	.068	.631
Triceps skinfold (mm)	.237	.097	.409	**.003**	−.252	.068
Subscapular skinfold (mm)	.046	.751	.462	**.001**	.042	.766
Human milk substitutes (*n* = 35^c^)						
Weight/age (z)	−.150	.397	.394	**.026**	−.097	.590
Body mass index (z)	−.162	.359	.429	**.014**	−.236	.186
Medium upper arm circumference (z)	−.022	.900	.348	.051	−.020	.913
Triceps skinfold (mm)	−.022	.900	.348	.051	−.020	.913
Subscapular skinfold (mm)	−.113	.523	.239	.188	.014	.937

Note

Bold indicates statistical significant value (*p* < .05) .

Values excluded due to technical problems in the sample handling or in the laboratory assay

a n = 5.

b n = 4.

c n = 3.

## 4 DISCUSSION

Ziegler (2006) has demonstrated that during the first 6–8 weeks of life, there is little difference in growth (gain in weight and length) between breastfed and formula‐fed infants, and that there are no consistent differences in adiposity during the first 4–5 months of life. Giannì et al. (2014) found that formula‐fed infants showed a different body composition through the first 4 months of life compared to breastfed infants, with higher fat‐free mass content. In addition, Gale et al. (2012) showed in a systematic review that in formula‐fed infants fat‐free mass was higher and fat mass was lower at 3–4 months than in breastfed infants. On the other hand, Bell, Wagner, Feldman, Shypailo, and Belfort (2017) have shown that formula‐fed infants gained weight more rapidly, which was out of proportion to linear growth, than did predominantly breastfed infants. These differences were attributable to greater accretion of lean mass, rather than fat mass; they concluded that any later obesity risk associated with infant feeding did not appear to be explained by differential adiposity gains in infancy.

Our data show that for infants at 8 and 16 weeks of life, most of the anthropometric indicators showed significant differences between the three types of feeding: FBF, PBF, and HMS. In addition, at these ages, the weight/age, weight/length, BMI indexes, MUAC, and skinfold indicators were significantly higher in infants receiving FBF than in those receiving HMS. Weight/age, BMI, and skinfolds were also found to be higher in infants receiving FBF versus PBF; FBF is likely to better protect infants with a greater increase in energy reserves during the first 6 months of life than infants fed a HMS. However, linear (cm) and cephalic (cm) growth only showed differences between these two types of feeding at 8 weeks of age. The infants who received PBF were a group that drew our particular attention because of their different anthropometric behavior. They were more affected in their anthropometric indicators than infants who received FBF but generally performed better in terms of growth and energy reserves than infants who received HMS (WHO, 2016).

Traditionally, PBF has been divided into three broad groups (Labbok & Krasovec, 1990): infants who receive <20% of a HMS (high breastfeeding); between 20% and 80% (medium), and those who receive <20% of human milk (low breastfeeding). From the point of view of nutritional requirements, it is difficult to give an adequate interpretation with this range of groups, especially infants receiving between 20% and 80% of human milk. This group of infants could be in a situation of nutritional vulnerability because the mother would not know exactly how much milk she produced and how much she should supplement with an HMS to avoid a potential risk of underfeeding or overfeeding her infant. It is clear that for an analysis of anthropometric and body composition indicators and the concentration of ARHs, a more detailed analysis of this type of breastfeeding would be required (Breij et al., 2017; Rao & Kanade, 1992; WHO, 2016; Yan, Liu, Zhu, Huang, & Wang, 2014).

In relation to ARHs, similarities and important differences were also observed between infants receiving FBF and those receiving PBF and HMS. In the case of ghrelin, a potent orexigenic, its concentration in infants appears to be more related to the need to be fed and nourished properly. The higher concentration of GLP‐1 and peptide YY is the result of regulatory mechanisms related both to body composition and to the infant's own growth needs (Vasquez‐Garibay et al., 2019).

Some of the observed results could be related to the physiological functions and mechanisms of action of these ARHs both in the gastrointestinal tract and in the CNS. It is known that the peripheral melanocortin 4 receptor (MC4R), which has an essential role in energy regulation, is implicated in the regulation of peptide YY and GLP‐1 (Breij et al., 2017; Choudhury, Tan, & Bloom, 2016). Peptide YY is a short peptide (36 amino acids) secreted by the neuroendocrine cells of the ileum and colon in response to feeding. It inhibits gastric motility; consequently, it increases the efficiency of digestion and nutrient absorption after a meal and increases the absorption of water and electrolytes in the colon. It seems obvious that the concentration of peptide YY, in particular, would have individual and important regulatory mechanisms in the infant who is in a crucial stage of accelerated growth (Breij et al., 2017; Perälä et al., 2013). Breij et al. (2017) have reported that breastfed infants have higher Peptide YY concentrations, which could be a link to the protective role against obesity in exclusive breastfeeding.

The peptide hormone GLP‐1 is 30 amino acids long, and its main source is the L‐cell of the intestine. This peptide is derived from the transcription of a gene called proglucagon whose physiological function is based on reducing blood glucose concentration through increased secretion of insulin and suppression of glucagon secretion by the pancreas (Meier et al., 2004; Schueler et al., 2013). Among its other functions, GLP‐1 inhibits gastric acid secretion and gastric emptying, and it suppresses food intake through the sensation of satiety. In the CNS, it increases the acquisition and strength of conditioned aversions to taste, anxiety, nausea, or visceral discomfort. In the presence of GLP‐1, the pleasurable value of food as well as the motivation (reward) for eating, and the amount and frequency of food consumption decrease (Graaf et al., 2016; Skibicka, 2013).

These assumptions become more relevant because when the correlations between anthropometric indicators and HARs are explored, a directly differentiated character is observed. For example, cephalic circumference as an indicator of brain growth is inversely and significantly related to ghrelin, while it correlates directly and significantly with leptin. Perhaps because there is a great need for brain growth in infants, there is a need for a higher concentration of ghrelin; this may be due to its role as an orexigenic hormone acting at key hypothalamic and midbrain circuits involved in feeding control to ensure adequate nutrient intake (Fidanci et al., 2010; Méquinion et al., 2013). However, direct and higher concentrations of leptin could be an indicator of the assurance of energy reserves that would promote brain growth. In contrast, lower overall growth and lower energy reserves to ensure growth would show lower leptin concentrations as a potential indicator of nutrient failure, food shortage, or chronic malnutrition resulting in the need for an allocation of energy reserves to ensure brain growth in particular (Kayardi, Icagasioglu, Yilmaz, & Candan, 2006; Stein, Vasquez‐Garibay, Kratzsch, Romero‐Velarde, & Jahreis, 2006; Yilmaz et al., 2005). The direct relationship of GLP‐1 with all the indicators that express energy reserves would have a similar interpretation to leptin, a situation that would be expected considering that both are anorexigenic hormones.

In contrast, it was evident that peptide YY, despite being an anorexigenic hormone, was not associated with any anthropometric indicators. This finding suggests that adiposity is not a factor that directly influences the peptide YY concentration and its potential function, and that the regulating mechanism of this hormonal biomarker could be influenced by appetite regulation of the infant. This is most probably related to signaling mechanisms of the gastrointestinal tract and their effect on the CNS. This argument is supported by the fact that in infants who received PBF and HMS only leptin correlated with energy reserve or adiposity indicators.

In addition, infants who received PBF also showed an inverse and significant relationship between ghrelin and cephalic circumference, which supports the need for these infants to boost their physiological need to eat (hunger) together with the need for hyperplastic brain growth. The CNS is a vital organ in the growth and development of human beings, and it surely has a direct relationship with growth hormones (insulin, ghrelin, growth hormone, and insulin‐like growth factor‐1 among others) (Gray, Meijer, & Barrett, 2014; Hara et al., 2014).

The main strength of this study was that it was a cohort of the mother–infant dyad identified in the immediate postnatal period, followed longitudinally along 4 months, and without the researcher's intervention on the type of feeding that mothers selected for their infants. One limitation of the study was that the number of infants fed HMS was lower than those who received FBF and PBF.

In conclusion, the main contribution of the study was the demonstration of the differences in anthropometric and body composition indicators among infants receiving FBF, PBF, and HMS at 8 and 16 weeks of postnatal age. In addition, there were marked differences in serum concentration of appetite‐regulating hormones in infants, especially in the highest concentrations of hormones related to increased food intake and growth, and lower concentrations of leptin, the hormone related to increase in the energy reserve. Finally, we observed the direct and often significant trend of linear correlations of most indicators of fat reserve with ghrelin, leptin, and GLP‐1, especially in infants who received FBF.

## CONFLICT OF INTEREST

All authors involved in these work disclose any potential sources of conflict of interest related to patent or stock ownership, membership of a company board of directors, membership of an advisory board or committee for a company, and consultancy for or receipt of speaker's fees from a company.

## ETHICAL APPROVAL

The recommendations of the Declaration of Helsinki were followed in its last Amendment during the 64th Annual Assembly organized by the World Medical Association, 2013.

Ethical review: This study does not involve any human or animal testing and was approved by the Committees of Bioethics and Research of the Hospital Civil Hospital of Guadalajara, and the Committees of Biosecurity, Bioethical and Research of the University of Guadalajara, Center of Health Sciences (CI‐01314).

Informed Consent: Written informed consent was obtained from the parents of the participating dyads.

Human testing (measurement of serum concentration of appetite‐regulating hormones) was necessary for our study.
